# “I’ll just deal with this on my own”: a qualitative exploration of experiences with self-managed abortion in the United States

**DOI:** 10.1186/s12978-021-01142-7

**Published:** 2021-05-04

**Authors:** Sarah Raifman, Lauren Ralph, M. Antonia Biggs, Daniel Grossman

**Affiliations:** Advancing New Standards in Reproductive Health (ANSIRH), University of California, San Francisco, 1330 Broadway Suite 1100, Oakland, CA 94612 USA

**Keywords:** Self-managed abortion, Abortion, Qualitative research, Missed-period pill, Abortion restrictions, Barriers to healthcare

## Abstract

**Background:**

A growing body of evidence indicates that some people seek options to terminate a pregnancy without medical assistance, but experiences doing so have largely been documented only among people accessing a clinic-based abortion. We aim to describe self-managed abortion (SMA) experiences of people recruited outside of clinics, including their motivations for SMA, pregnancy confirmation and decision-making processes, method choices, and clinical outcomes.

**Methods:**

In 2017, we conducted 14 in-depth interviews with self-identified females of reproductive age who recently reported in an online survey administered to Ipsos’ *KnowledgePanel* that, since 2000, they had attempted SMA while living in the United States. We asked participants about their reproductive histories, experiences seeking reproductive health care, and SMA experiences. We used an iterative process to develop codes and analyzed transcripts using thematic content analysis methods.

**Results:**

Motivations and perceptions of effectiveness varied by whether participants had confirmed the pregnancy prior to SMA. Participants who confirmed their pregnancies chose SMA because it was convenient, accessible, and private. Those who did not test for pregnancy were motivated by a preference for autonomy and felt empowered by the ability to try something on their own before seeking facility-based care. Participants prioritized methods that were safe and available, though not always effective. Most used herbs or over-the-counter medications; none used self-sourced abortion medications, mifepristone and/or misoprostol. Five participants obtained facility-based abortions and one participant decided to continue the pregnancy after attempting SMA. The remaining eight reported being no longer pregnant after SMA. None of the participants sought care for  SMA complications; one participant saw a provider to confirm abortion completion.

**Conclusions:**

There are many types of SMA experiences. In addition to those who pursue SMA as a last resort (after facing barriers to facility-based care) or as a first resort (because they prefer homeopathic remedies), our findings show that some individuals view SMA as a potential interim step worth trying after suspecting pregnancy and before accessing facility-based care. These people in particular would benefit from a medication abortion product available over the counter, online, or in the form of a missed-period pill.

## Plain language summary

Some people in the United States (US) attempt to end a pregnancy on their own without medical supervision. What we know about this experience comes from studies focused on people who go to clinics. In this study, we conducted 14 interviews with self-identified women ages 18–49 who recently reported attempting to end a pregnancy on their own and who were recruited outside of the clinic setting. We asked participants about their fertility histories, experiences seeking reproductive health care, and experiences ending a pregnancy without medical assistance. Those who took a pregnancy test and then chose to end the pregnancy on their own did so because it was convenient, accessible, and private. Those who did not test for pregnancy felt empowered by the ability to try something on their own before seeking facility-based care. All participants prioritized methods that were safe and available, though not always effective. After they attempted to end the pregnancy on their own, five participants accessed abortion care in facilities, one decided to continue the pregnancy, and eight were no longer pregnant. Our findings show that, in addition to people who end a pregnancy on their own as a last resort (after facing barriers to facility-based care) or as a first resort (because of preferences for homeopathic methods), a third group values having an interim step to try after suspecting pregnancy and before accessing facility-based care. These people would particularly benefit from a medication abortion product available over the counter, online, or in the form of a missed-period pill.

## Background

In the United States (US), a growing body of evidence suggests that some people seek options to terminate a pregnancy outside of the formal healthcare system. Estimates of the prevalence of self-managed abortion (SMA) in the US come primarily from studies of people seeking care in abortion clinics or other primary or reproductive health care clinics. These studies found that between 2% and 13% of people accessing facility-based care reported having taken or done something to end a pregnancy on their own without medical assistance [1–4]. Guttmacher’s 2014 survey of patients accessing facility-based abortion found that 1.3% and 0.9% reported ever having taken misoprostol or other substances, respectively, without guidance of a healthcare provider, to terminate a pregnancy [5].

However, these estimates may not capture the experiences of people who never access facility care. In 2015, a population-based study conducted among reproductive-age women in Texas found that 1.7% reported having ever self managed an abortion [6]. Two years later, cognitive interviews about SMA conducted in four states provided more context for how people interpret questions about SMA and informed the language used in the first nationally representative study of SMA prevalence [7]. This study concluded that 7% (95% CI: 5.5–8.4%) of self-identified women in the US will attempt SMA at some point in their lives [8]. The prevalence of SMA may be even higher in certain subgroups of the populations; a recent study exploring abortion attempts without clinical supervision among transgender, nonbinary, and gender-expansive people in the US found that 36% and 19% of ever-pregnant study participants reported considering and attempting SMA, respectively [9]. Evidence suggests that SMA occurs across the US in both states with restrictive and supportive abortion policy landscapes [8, 10]. Yet as barriers to facility-based abortion services increase across the country, SMA may become more prevalent. Abortion providers report caring for a growing number of SMA patients in the last 5 years [11, 12].

Individual motivations for attempting SMA vary widely. They include fear of abortion stigma, a preference for privacy and autonomy, receiving recommendations for informal methods from family or friends, and logistical challenges to accessing facility-based care such as lack of transport or funds [2, 10, 13, 14]. People who are young, less knowledgeable about abortion legality, and/or identify as transgender, nonbinary, or gender-expansive are some of those shown to be particularly interested in SMA [2, 9, 15]. For the latter group, concerns about accessing facility-based care may be related to intimate partner violence, refusals of care from providers, and barriers to insurance coverage tied to gender markers [9].

Local abortion policy context and the availability of nearby abortion providers may also influence one’s decision to try SMA. A disproportionate majority (76%) of the 6022 requests for abortion medications from US residents to an online telemedicine service between October 2017 and August 2018 were from abortion hostile states; 58% of all women 15–44 years old in the US lived in those states [16]. In some states in the South and Midwest, the proportion of reproductive-age women living in counties without an abortion provider is as high as 90% [17]. In Texas, shortly after half of abortion clinics closed due to the passage of House Bill 2 in 2013, abortion patients were confused about where to go for care and concerned about potential increases in cost and travel time associated with obtaining in-clinic care; five out of 23 patients interviewed said they considered SMA after the clinic closures [3]. During the COVID-19 pandemic, when access to facility-based care was even more limited, there was a 27% increase in the rate of online requests for self-managed medication abortion across the US [18].

For the most part, studies of experiences with SMA, both quantitative and qualitative, have included samples of abortion patients recruited in clinic settings, people seeking abortion online, or people seeking general health care in clinic settings [1, 2, 10, 13]. One recent study included qualitative interviews with participants recruited from community settings in Texas [14]. There is growing interest in and need for updated information about motivations for and experiences with SMA among people who may not present for care at facilities. The purpose of the present study is to better understand the details of people’s experiences with SMA, including all method types, among a US sample of reproductive-age self-identified women. We explored motivations, sources of information, decision-making processes, method choices, clinical outcomes, and overall recommendations of individuals who attempted SMA while living in the US.

## Methods

We conducted in-depth qualitative interviews with people of reproductive age who reported having ever attempted ending a pregnancy on their own without medical assistance. Study participants were initially recruited in August 2017 as part of a quantitative survey [8]. Eligible survey respondents were self-identified females, 18–49 years old, who spoke English or Spanish, were living in the US, and were enrolled in the *KnowledgePanel*, a nationally representative online panel administered by the market research company Ipsos [19]. Additional eligibility criteria for participation in the interview portion of the study included having reported prior experience with SMA in the survey. Details about the survey and sampling methods are published elsewhere [8, 19]. Participants eligible to participate in an interview portion of the study were asked at the end of the survey whether they would be interested in participating in a follow-up interview about their SMA experience(s); those interested were re-contacted by Ipsos to confirm interest and contact information and selected for an interview using convenience sampling. Ipsos shared interested participants’ contact information with study researchers, who contacted participants no more than three times to schedule a telephone interview. Those who answered yes to the following question were considered to have prior experience with SMA:

“As we mentioned earlier, some women may do something on their own to try to end a pregnancy without medical assistance. For example, they may get information from the internet, a friend, or family member about pills, medicine, or herbs they can take on their own, or they may do something else to try to end the pregnancy. Have you ever taken or used something on your own, without medical assistance, to try to end an unwanted pregnancy?”

We excluded people who had only attempted SMA outside of the US given potential differences across countries regarding the legal and social context of abortion and SMA. We also excluded people who reported SMA attempts that occurred prior to the year 2000 in order to reduce recall bias. In 2000, the FDA first approved mifepristone for use in the US and the first paper documenting post-Roe SMA in the US was published [20], both of which distinguish the post-2000 era from the prior period with regard to SMA. Finally, we excluded attempts where the only method used was emergency contraception pills (EC) prior to the confirmation of pregnancy because it was unclear whether these participants had used EC appropriately to prevent implantation after sexual intercourse (and simply thought this was considered an abortion) or whether they had used it to attempt to terminate a pregnancy. This analysis was intended to specifically focus only on attempts to terminate a pregnancy.

The interviewer confirmed before proceeding that the participant was in a private location. The interview was semi-structured, allowing for extensive interviewer probing. The interview guide included questions related to participant access to and use of healthcare services in general and reproductive health care specifically, participant’s pregnancy history and personal experience(s) with SMA, including the discovery of the pregnancy, reasons for pursuing SMA, sources of information about SMA, methods used, side effects, clinical outcomes, and reflections on the experience overall. We designed the guide to elicit detailed information about the SMA experience from pregnancy discovery to pregnancy outcome. We used participants' most recent SMA attempt to summarize methods and outcomes in this manuscrcipt. Participants were reimbursed $50 through Ipsos’ points program.

One researcher (SR), who is female, employed by the research study as a project director, and formally trained in qualitative interviewing and analysis, contacted all participants and conducted all of the interviews after confirming eligibility and requesting verbal consent for the interview and audio recording. The research team did not have established relationships with participants prior to the study. Participants were informed that the study aimed to learn more about their previously reported SMA attempt. The interviewer took notes during interviews and wrote a brief summary of each interview within 1 h after its completion. A professional transcribed all interviews, and the interviewer reviewed all transcripts to ensure validity and redaction of identifying information. The transcribed interviews were read and re-read by SR and DG; participants did not review transcriptions. SR developed a codebook based on themes identified in advance. She applied codes to a subset of transcripts, identified additional themes in the data during this process, discussed these themes with the study principal investigator (DG), and then revised the codebook accordingly. After finalizing the codebook, SR applied final codes to all transcripts using Dedoose Version 8.3.17, a web application for managing, analyzing, and presenting qualitative and mixed methods data [21]. Coding was not reviewed with participants. In the analysis phase, SR created an Excel database describing SMA attempts identified in the interviews, including whether the participant tested for pregnancy, the methods they used, their experience after using the method, and their pregnancy outcome, and analyzed transcripts using inductive thematic content analysis methods for qualitative research [22]. We generated frequency counts of themes identified and compared trends in these themes to the established literature. Participant quotations are presented here to illustrate the themes and findings from the analysis. In the results, the following participant information is included in parentheticals: participant age at time of the most recent SMA attempt and state of residence at the time of the most recent SMA attempt. All study activities were approved by the UCSF Institutional Review Board. The reporting of this study follows the Consolidated criteria for reporting qualitative research (COREQ) checklist [23].

## Results

**Fig. 1:**
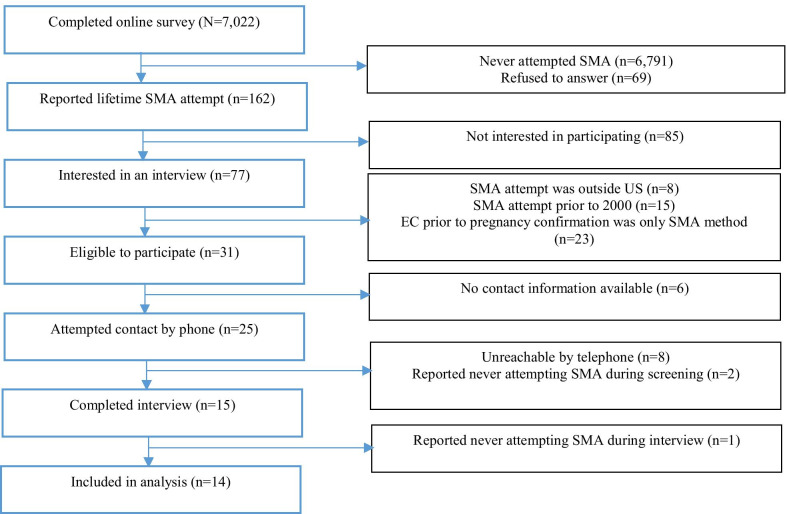
Study sample. *EC* emergency contraception, *SMA* self-managed abortion, *US* United States

Eligible participants were identified via the administration of an online survey with a nationally representative sample of self-identified women ages 18–49. Of the 7022 participants who completed the online survey, 162 reported an SMA attempt; 77 of those indicated interest in a follow-up interview (Fig. 1). Of these 77, we excluded respondents who only reported attempting SMA before the year 2000 (n = 15), living outside of the US at the time of the SMA attempt (n = 8), and taking EC prior to confirming pregnancy as their only method of SMA (n = 23). Ipsos provided telephone and/or email contact information for 25 of the remaining 31 eligible individuals, based on availability of information in Ipsos’ databases. Eight of the 25 were unreachable. When only an email address was available, the study team emailed the participant and requested to schedule a telephone call to confirm eligibility and interest in an interview. Of the 17 we spoke with by telephone, three were determined ineligible after reporting no prior SMA experience during the pre-screening (two participants) or during the interview (one participant). The SMA methods reported by these three respondents in the previous quantitative survey included misoprostol, some other drug, and/or physical harm. It is unclear if these three participants incorrectly completed the survey, or if they felt uncomfortable talking about their SMA experience during the telephone interview. In total, 14 participants were included in the analysis.

All interviews were conducted by telephone in English, included only the participant and the interviewer, and varied in length from 30 min to 1 h None of the participants requested to complete the interview in Spanish. Due to an audio-recording error, six of the interviews were repeated; we were unable to repeat one interview and therefore have a detailed summary of the interview but no transcript. The interviewer (SR) has no reason to conclude this interview was substantively different than the rest of the sample.

The study sample had a median age at interview of 30.5 years old (interquartile range: 26–35.5) and a median age at latest SMA attempt of 24.5 (interquartile range: 19–31). Twelve participants were non-Hispanic white and two were non-Hispanic Black (Table 1). At the time of the interview, all but one participant had at least some university-level education, most participants were employed, and five were living below 100% of the federal poverty level. About one-third of the sample was married or living with their partner, one-third was divorced or separated, and one-third was never married. Eight of 14 participants were nulliparous, six had a previous abortion and only two reported experience with medication abortion. Though gender identity was not explicitly asked of participants, one respondent self-identified as “gender fluid.” Some participants said they had attempted SMA more than once (noted by an asterisk in Table 1). In describing their most recent SMA attempt, it was clear that some participants with multiple experiences talked about them collectively.

**Table 1:** Summary of participants’ most recent self-managed abortion attempts

ID	State of residence at most recent SMA (current state of residence, if different)	Race/ethnicity	Approximate year of most recent SMA	Approximate age at most recent SMA	Pregnancy test prior to SMA	SMA method	Outcome
1	Arkansas	Non-Hispanic white	2014^a^	23	No	3 cups parsley tea, 2000 mg vitamin C, 1 cup of chamomile tea daily for 2–5 days	Negative home pregnancy test after SMA
2	California	Non-Hispanic white	2010	15	Yes	Took 3–4 oral contraceptive pills at once	Negative pregnancy test at clinic after passing the pregnancy
3	California	Non-Hispanic white	2017	36	Yes	Drank 2 smoothies per day (including 5 10 mg tablets of cinnamon, 5 2000% daily value vitamin C tablets, a whole papaya, and half a pineapple) over 2 weeks	Clinic abortion
4	California	Non-Hispanic white	2011	37	No	Vitamin C tablets over 1–5 days	Return of period
5	Florida	Non-Hispanic white	2015	25	Yes	6000 mg vitamin C and 3–4 capsules of dong quai daily for 1.5 weeks	Clinic abortion
6	Illinois	Non-Hispanic Black	2017	26	Yes	Took 3–4 ibuprofen and antibiotics pills a few times over several days (up to 1 week)	Clinic abortion
7	Louisiana	Non-Hispanic Black	2004^a^	24	Yes	4–10 Humphrey’s 11 menstrual regulation pills daily for about 4 days	Clinic abortion
8	Maryland (Texas)	Non-Hispanic white	2008^a^	22	No	Liquid drops of black cohosh and rosehips herbs daily for 4 days	Return of period
9	Ohio	Non-Hispanic white	2015	32	No	4 pills vitamin C daily for 2 days, 2 tablespoons of gingerroot on first day	Miscarriage/return of period
10	Ohio	Non-Hispanic white	2014	18	Yes	Drank vodka over 3–4 h	Negative home pregnancy test after SMA
11	Ohio	Non-Hispanic white	2013^a^	27	No	Took 2 vitamin C (2000% DV) pills, drank parsley tea by steeping it in hot water, inserted parsley leaves in her vagina over 3 days, replacing the leaves regularly	Return of period
12	South Carolina	Non-Hispanic white	2013	35	Yes	Inserted a long plastic spoon into her vagina	Continued pregnancy
13	Washington (California)	Non-Hispanic white	2003	18	Yes	Drank liquor, cough syrup, and over-the-counter Dramamine	Clinic abortion (was told embryo was non-viable)
14	Washington	Non-Hispanic white	2005	18	Yes	Antibiotics, Mountain Dew, ibuprofen, caffeine pills, alcohol	Miscarriage/return of period

^a^Participant reported more than one self-managed abortion attempt. The most recent attempt is included in the summary

### Reasons for attempting SMA

Reasons for attempting SMA varied based on whether or not the respondent had a confirmed or a suspected pregnancy. Among the six respondents who confirmed that they were pregnant using an at-home pregnancy test, a primary reason for attempting SMA was a lack of knowledge about where to access facility-based abortion or how to obtain financial support for abortion services. All participants seemed to be aware that abortion was legally available, but not all knew where they could go to request an abortion, the gestational age limitations on abortion in their state, or how to seek financial support for covering the cost of the abortion. A respondent who had a positive pregnancy test prior to attempting SMA said, “I do not know where you could go to get an abortion if you wanted to get one. I have no idea. It’s my understanding that it would be self-pay, because Medicaid does not pay for it. Every insurance that I’ve ever had has said that they don’t pay for it” (35, South Carolina). This participant ultimately carried the pregnancy to term. Another participant, who had a positive pregnancy test prior to SMA, said she made the decision to pursue SMA based on what was ultimately inaccurate and insufficient information. She chose to follow the advice of someone who she believed had experience with unplanned pregnancies, “Instead of doing what my adoptive mom had taught me and go to Planned Parenthood or to her…I went to my biological mom instead because I felt like she had more direct experience with unplanned pregnancies…” (15, California). Later she said, “I wish sex ed in high school and resources in the community had been better advertised…better quality.”

For some participants, perceived stigma about abortion combined with a desire for privacy influenced their decision to seek SMA as an alternative to facility-based care. One participant who was employed by the Navy explained that she had assumed that seeking abortion services through her employer’s health insurance was not an option and she did not feel comfortable asking for further information. When asked whether abortion services were available through the Navy, she replied:

“I've never asked. But I don't know of any of my friends throughout the years that … I could have asked. And I didn't really want to divulge that information and have to answer any questions. So…I kind of just left it alone. I was like, ‘I'll just deal with this on my own’" (36, California)

Others mentioned fear of feeling judged or shamed by others at the clinic as well as perceptions that clinicians would not be able or willing to help them. One participant said she didn’t consider going to a clinic in part “because I knew that …I would have had to tell somebody else about my stupidity of allowing myself to become pregnant” (35, South Carolina). Recalling a previous clinic experience, which partially motivated her to try SMA, one participant explained, “There's people there that stand outside with these boards. Of course, they're against abortion…that was probably about the worst part, and just kind of feeling ashamed doing it” (25, Florida). Another said she attempted SMA after confirming her pregnancy because she was concerned that her doctor would try to further persuade her not to have an abortion. She explained that when she requested an abortion prior to attempting SMA, she was told to continue the pregnancy:

“I didn’t think they [would] help me with the abortion. So I took it out on myself and just did some stuff on my own… Because they just wanted me to carry the baby full term… [The clinician] didn’t really give me no options to be honest. … I was telling her everything and the only thing that she was saying [was], ‘well, it would be best if you could just carry the baby the full nine months’” (26, Illinois)

Other barriers to facility-based abortion care for participants with a confirmed pregnancy included logistical constraints, such as concern about delays due to the time required to find a clinic and schedule an appointment and the cost and time required for travel to a clinic. One participant said, “I didn’t go to a clinic just because of price and time…. I don’t have a car. It was the dead of winter, which makes getting around here kind of annoying. I’d have to take a bus out somewhere…” (32, Ohio). Another said that lack of transportation and available appointment times, combined with a desire not to repeat prior negative abortion experiences, contributed to her desire for SMA. After confirming her pregnancy and attending an initial appointment, she was not able to find an appointment for the abortion for another 2 weeks, so she decided to try SMA (36, California). Another participant explained that she had little knowledge about abortion clinics, most of her family and friends were anti-abortion, and she did not feel at the time like she could access care due to logistical and financial barriers. She said, “It was hard to find. There wasn’t very many clinics around me to begin with. The ones that were [there] were just so expensive, the procedures themselves, that the cost and just availability of people wasn’t very good” (18, Ohio).

Those who attempted SMA after suspecting but not confirming a pregnancy also reported prioritizing privacy and being nervous to admit to anyone (including themselves) that they might be pregnant. In addition to these reasons, those who did not confirm the pregnancy with a pregnancy test explained that being early on in the suspected pregnancy and having a preference for herbal medicines and a dislike for going to health facilities influenced their decision to pursue SMA. Several participants explained they felt most comfortable trying alternative methods for SMA during a grey area when they suspected but had not confirmed a pregnancy. One respondent explained why they opted to take vitamin C after a missed period:

“…because it was so early, I needed a way to calm myself down and … put it out of my mind and not stress about it unduly. So, yeah, just being able to take these tablets and say we're doing this - we're doing something. We're not just sitting on our hands and ignoring the problem…emotionally it felt comforting to be doing something, even though I didn’t 100 percent believe that it would be effective, but it was something to do while I waited and just to kind of feel slightly empowered even if I was aware that it was a false empowerment” (37, California)

Another participant drank parsley tea, inserted parsley into her vagina repeatedly over several days, and took vitamin C pills. Despite knowledge of available facility-based care, she saw SMA as a way to maintain privacy and assumed that herbal methods would only be effective early on in pregnancy. She said,

“…[SMA] seems like a pretty good go-to without having to get anybody involved or anything…it seemed like a… safe and kind of natural way of going about it, so I figured I really didn’t have anything to lose, especially [compared to] seeing a doctor or whatever… I think it would be safe to try it at any step, but I think it would only work very early on [in pregnancy]… if it’s over a month late I’d also see a doctor …If I tried this first step for a week and it didn’t work, then I would take a pregnancy test…” (27, Ohio)

Some participants explained further why they decided not to take a pregnancy test immediately when they suspected a pregnancy. For some, the ambiguity allowed them to be more comfortable with the decision to attempt SMA and afforded them more time to consider their options if they were pregnant. One participant said she “did herbs to make sure that I didn’t get pregnant, or that I wasn’t pregnant.” She explained further:

“I kind of didn’t want to know [if I was pregnant], because that would be an abortion, right? And I hadn’t actually had an abortion up until that point, and I hadn’t been faced with having to make a real choice …if you don’t know and it’s really early on and you took these herbs, then is it really an abortion? It’s almost like a grey area, right? The adult in me is like, of course it’s still an abortion, but it makes you feel better, I guess” (22, Maryland)

Another respondent said, “I think it was also a fear that if I go buy a pregnancy test, am I basically admitting that I might be pregnant…? So it just felt kind of…official” (23, Arkansas). This same participant later reflected, “If I had had a positive pregnancy test, I would have been on the phone making an appointment with Planned Parenthood right away” (23, Arkansas). Similarly, another participant said, “If I definitely knew I was pregnant, that might have made me just go to Planned Parenthood and find out how to take care of it. I might have taken things more seriously, I guess…” (32, Ohio). She later added, “… thinking ‘I don’t know if I’m pregnant or not’ and doing something, and then you’re not pregnant, it’s like, well you’re sort of dodging the bullet, whereas being like, ‘okay, I am deliberately ending a pregnancy’ is sort of…taking on a lot more” (32, Ohio).

### SMA method

About half of participants attempted SMA with herbs, including vitamin C, parsley, Dong Quai, rose hips, gingerroot, chamomile, and black cohosh. Others took medications, such as analgesics, antibiotics, oral contraception, menstrual regulation pills, and caffeine pills. No one in the sample reported using mifepristone or misoprostol. Only one participant attempted to terminate the pregnancy via intrauterine trauma, by inserting a long plastic spoon into the vagina. Though all participants reported that they attempted SMA in their initial survey responses, in interviews some referred to their use of these methods as inducing a period or miscarriage, rather than abortion.

Most respondents said their primary source of information on SMA methods was the internet. One participant explained, “I read and cross-referenced multiple [websites] so that I was getting the best information on it I could” (25, Florida). Another respondent explained: “I tend to compare a lot of different sources of information and sort of see what correlates and what doesn't…if one said take five and one said take two, I probably would have been, okay, so three's good… just sort of aim up the middle” (32, Ohio).

Some participants reported learning about SMA methods from their partner or friends. For example, one respondent decided to try Humphrey’s 11, a natural remedy used for menstrual regulation, because her partner’s sister had successfully used it to bring her period back. Another took multiple birth control pills simultaneously based on her mother’s advice. On the other hand, most said they did not consult anyone about the experience, including about their method choice. One participant said, “I was just doing it on my own. I didn’t hear it from nobody, but I knew what I had to do” (26, Illinois). Another respondent explained why she didn’t talk to anyone, “I guess I just didn't feel like I needed to reach out to anyone. I just felt like I had the situation under control” (32, Ohio)

When deciding which SMA method to use, most participants prioritized safety over other factors. Several said they dismissed methods they had heard were potentially dangerous, such as pennyroyal, coat hangers, or bleach. One participant said the following about her decision to take menstrual regulation pills: “I didn't want to die. So I was like, don't mix up things that you really don't know anything about” (24, Louisiana). Another, who chose to use parsley and chamomile tea and vitamin C, explained:

“Yeah, I definitely wasn’t about to do anything that could kill me, especially since I knew that I could go to Planned Parenthood and get a medication abortion that would be safe if I turned out to actually be pregnant” (23, Arkansas)

Another said, “I was like, well, I'm not going to do [bleach] because I don't think I could take that and actually be okay at the end. And then I just saw the alcohol. And I was like that sounds like a way that at least wouldn't really harm me so much but would get what I wanted to happen” (18, Ohio). The participant who inserted a long spoon into her vagina indicated that she was not concerned about safety because she did not intend to have more children: “it was my theory that if I somehow damaged myself, I didn’t want to have any more children anyway, so therefore the worst-case scenario would be that they would give me a hysterectomy and that wouldn’t be an issue anymore” (35, South Carolina).

In addition to safety, participants also considered availability, effectiveness, and potential legal risks when choosing an SMA method. One participant explained, “the vitamin C and ginger were definitely one out of convenience because they were things that I had. And even if I didn't have them, I could get them at the grocery store rather than some exotic herb or something I would have to order or go out and get” (32, Ohio). Some participants, in search of an effective method, looked specifically for substances that were contraindicated for pregnancy. One respondent said, “So I kind of verified it against WebMD. Because …it said things that you kind of should avoid when you're in early pregnancy… So I just kind of used all of them” (36, California). Another agreed with this sentiment, “So I feel like anything that they tell you not to do if you’re pregnant, if you don’t want to be pregnant, you should do that” (23, Arkansas). In assessing the possibility of sourcing medication abortion pills outside of the formal health care system, this participant also raised concerns about legal risks:

“… and there's always getting the abortion medication on the black market or trying to order it from other countries, but I didn't really look into that too heavily because I would have been afraid of getting caught and I, you know, you can get prosecuted for buying drugs illegally and stuff like that” (23, Arkansas)

## SMA outcomes

**Fig. 2:**
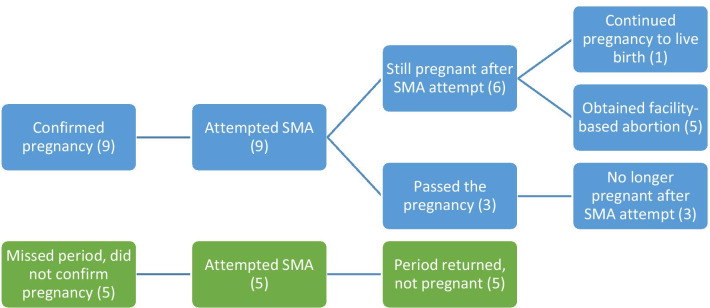
Pathways from pregnancy suspicion or confirmation to outcome

Figure 2 presents pathways from pregnancy suspicion or confirmation to pregnancy outcome for the 14 participants. Nine participants reported having a positive pregnancy test, all in the first trimester of pregnancy and prior to attempting SMA. Three of these participants believed the SMA attempt was a success and they reported that they had “passed” or “lost” the pregnancy and that menstruation returned. One said that, after her positive pregnancy test, she took ibuprofen and caffeine pills, antibiotics, and alcohol and then bled for 3 weeks (18, Washington). Another said she drank vodka over several hours after seeing her positive pregnancy test, then experienced cramping, “passed the pregnancy” in the bathroom, and confirmed she was no longer pregnant with a negative pregnancy test (18, Ohio). A third participant took multiple oral contraceptive pills and 2 or 3 days later she experienced severe cramping, which lasted a couple of hours. She went to the bathroom, vomited a couple of times, and then passed the pregnancy (“debris came out…I saw it, and thought that’s more than just blood clot”). She saved the pregnancy and took it to her primary care physician the next day who confirmed that “it looked like a fetus” at roughly 2 or 3 months’ gestation (15, California). This participant did not receive any treatment during the visit, and none of the other participants reported seeking medical care related to the SMA attempt.

Six of the nine participants who had a positive pregnancy test prior to attempting SMA said their SMA attempt was not successful. They noticed no effects or changes in pregnancy symptoms over 1 or 2 weeks after the SMA attempt. One decided to continue her pregnancy, and the remaining five obtained a facility-based abortion. One participant, in the latter group, did question whether her attempts to end the pregnancy (using alcohol, cough syrup and Dramamine over a 2-week period) may have had some effect when she was told by clinicians that the fetus was no longer viable (18, Washington).

The remaining five participants attempted SMA without taking a pregnancy test to confirm whether or not they were pregnant. These participants said that they had missed one or two periods (and were therefore likely in the first trimester if pregnant) at the time they decided to attempt SMA. All of these participants said that their period returned after they attempted SMA, but all were also unsure whether or not this was due to the SMA method they used. A couple of the respondents assumed that their SMA attempt was likely ineffective and that they had not been pregnant in the first place. One said, “It was a good psychological tool for me. It was good self-psychological care. It successfully allowed me to not obsess about it. And as for whether it was effective in bringing about my period, no idea, although I suspect not” (37, California). Another explained, “I’ve always pretty much assumed that it was probably the case that I was not pregnant. I guess I have no way of verifying for sure whether the steps that I took actually had, you know, an effect, so I guess I couldn’t say for sure” (23, Arkansas).

### Reflections on SMA

Participants’ overall reflections about their experience with SMA, and whether they would recommend SMA to a friend or someone else who found themselves in a similar position, varied depending on whether they believed the method had been successful and whether they had confirmed the pregnancy. Those who concluded that the method had been ineffective generally said they would not use the same method again in the future but reserved the possibility that it might still be effective for others. One participant said she would not recommend taking ibuprofen and antibiotics because it was “a stupid decision” and ineffective (26, Illinois). Another participant, who took vitamin C and dong quai, said she would not personally try it again because it was not effective for her, yet she would recommend that others try it in case it works for them (25, Florida). She said, “Now, I know that [the herbal method] didn’t work for me before. Would I do it again? Probably not. I probably wouldn’t waste my time with trying it.”

Those who were not sure whether their SMA attempt had worked recommended trying both SMA and facility-based options. For example, one woman who took herbs to bring her period back explained: “I would probably share what I knew and what I had done and you know I would definitely stress that it would not be the *only* step to take if she didn’t want to be pregnant, but that it was something that she could do” (23, Arkansas). Another participant said that, even though it was ineffective, she would recommend it as a first step before seeking care at a clinic; she said, “I kind of feel like it doesn’t hurt to try. It’s not like I was taking anything that would harm me. …if it doesn’t work, then, you know, you’ve always got Planned Parenthood as like a fallback plan” (36, California). One participant said she would advise speaking with a provider before trying SMA because she was frustrated not to have had more information about her options beforehand; she said about her decision to try SMA, “it was dumb, it was an ill-informed decision. I wish that I had been better informed. I wish that I’d asked for help from other sources as well” (15, California).

Of those who ended up seeking facility-based abortion services, most preferred their SMA experiences over going to the clinic and still wished they could have avoided the clinic. One explained that she wished her SMA attempt had been successful because it would have saved her time, money, and “having to feel like—I don’t know—shame and kind of like just the whole dread of sitting in an office, waiting for this thing to take place” (36, California). Another participant said she preferred her SMA approach because it was less physically painful and more private compared to her facility-based abortion:

[The herbs] did not hurt like the other medications did. And, also, just the general environment of going to an abortion clinic, it - I don't know if they just can't have sympathy or empathy or they don't want to, or it's just a source of income… if you don't have someone there to cry with, like, you will be alone....And there's more privacy to it, too. There's more privacy to going to the store and buying herbs. Or, even now, you can have it shipped to your house, even more privacy. Having to go through a clinic, like, half the time, they have people picketing outside” (22, Maryland)

Another participant explained that the facility-based care she received was effective and efficient, but that it was also impersonal and implied that she would have preferred more individual support:

“The care's good. It's easy to make appointments. People are friendly enough. But, things are a little bit - I mean, clinical isn't really the right word … But, just impersonal because, I mean, it's not always the same doctor. You don't really develop a rapport with anyone. They're just sort of about, okay, you want birth control. Here's what you have to do. Fill out these forms. You know…they provide the care you need. But they're really just about just that, just getting you in and out, kind of” (32, Ohio)

One participant who received a facility-based abortion agreed that the experience was relatively impersonal, but that in the end she appreciated the efficiency of the process. In reflecting on her experience, this participant weighed the potential effectiveness and potential harm to the fetus of her SMA approach and concluded that she should have simply gone to the clinic in the first place. She explained:

“I wish I'd been able to just go [to the clinic] to begin with. Because, even if it was impersonal and, you know, maybe somewhat awkward, as well, it was just in and out. Like really quick… Knowing what I know now, the chances of [her SMA method] actually ending a pregnancy are so unbelievably minute, compared to the damage it could actually do to the fetus…if I hadn’t had the miscarriage or if I hadn’t had an abortion, then would I have destroyed this poor person’s life because I’d done all these things trying to end this pregnancy that I did not want?” (18, Washington)

Overall, most participants chose to use SMA methods that were relatively harmless and likely to be ineffective at ending a pregnancy. As a result, they viewed the method as worth trying in case it had the intended effect but also not a sufficient approach that one could count on nor something they would necessarily recommend to others, given uncertainty about effectiveness.

## Discussion

Results from this study offer new evidence into the methods used and reasons motivating SMA among reproductive-age people living in the US, which appear to depend on where they are in the process of pregnancy suspicion and confirmation. Those who confirmed they were pregnant with home pregnancy tests sought SMA because of privacy concerns and barriers to facility-based abortion, including cost, time, and emotional stress related to abortion-seeking at clinics [24]. Those who suspected but did not confirm a pregnancy chose SMA because it was empowering to be able to do something to regulate their menstruation and/or end a potential pregnancy during a time of uncertainty. It was also comforting to remain unsure about whether what they experienced would be considered an abortion. Not knowing for sure if they were pregnant made it impossible to be certain about whether their actions were responsible for terminating a pregnancy.

SMA is often discussed as either a last resort, pursued by individuals who lack access to facility-based care, or as a preference over facility-based care, chosen by people who generally dislike medical facilities or allopathic medicine in favor of natural or homeopathic remedies [10, 25]. The results of this study suggest that there is a third type of individual who pursues SMA: those who suspect but have not confirmed a pregnancy and seek potential solutions to try in the interim after a missed period to see whether their period will return before actively pursuing facility-based care. These individuals did not necessarily view their actions as an attempt to end a pregnancy, but usually as an effort to regulate or bring back their menstruation. The existence of these individuals, who do not necessarily view their experiences as SMA, should be an important consideration in efforts to accurately estimate the prevalence of SMA and the extent to which SMA may be underreported in research.

Other qualitative research indicates that some people view SMA as something other than abortion, in part because it can be done so early in pregnancy [2]. Evidence demonstrates that people in the US would be interested in a medical menstrual regulation option, or a missed-period pill, in order to avoid knowing if they terminated a pregnancy and to minimize internal and external abortion stigma [26]. Similarly, some participants from the present study who attempted SMA without pregnancy testing said their personal views about abortion played a role in their decision to try SMA and they notably referred to their experiences as inducing a period or miscarriage rather than an abortion. These patterns further highlight that abortion stigma plays an important role for some who consider and attempt SMA. Those who seek SMA not as a preference or as a last resort but rather as an interim solution may be interested in having access to both self-managed *and* facility-based abortion services at different times throughout a pregnancy experience, depending on where they are in the process of suspecting and confirming a pregnancy and making the decision to terminate.

Our findings about reasons for seeking SMA resonate with results from prior evidence of motivations for seeking SMA, including the cost of facility care, clinic access barriers, recommendations from friends or family, preferences for self care, and stigma [2, 10, 14]. Our findings about motivations for SMA-seeking also support conclusions from a study investigating interest in and support for alternative models of medication abortion service provision (over the counter, online, or advance provision access). Biggs et al. found that 49% of adult women of reproductive age living in the US supported at least one of these three models, and 30% were personally interested in one or more of them because they afforded individuals more privacy, convenience, and the ability to end a pregnancy earlier in gestation [19]. These alternative provision models may be of interest in particular to those interested in SMA as they would allow people to end their pregnancies outside of the clinic setting safely and effectively, earlier in pregnancy, and with more privacy and autonomy than may be currently available.

The majority of SMA methods reported in prior studies have been readily accessible and generally thought to be ineffective for pregnancy termination. These include over-the-counter medications (such as pain relievers), herbs, alcohol or drugs, and physical manipulation [1, 2, 25]. Some people have obtained mifepristone and/or misoprostol from online sources or in marketplaces outside of the formal health system to induce abortion with seemingly higher likelihood of completion [2, 27, 28]. In the present study, most participants prioritized accessibility and affordability when choosing an SMA method, which led them to primarily try herbal remedies and over-the-counter medications and substances, which were available at home or in nearby stores. None of the participants attempted to obtain mifepristone or misoprostol for SMA despite their stated interest in safety and effectiveness.

One participant highlighted concern of potential legal risks when choosing an SMA method. Reports from legal experts confirm that those who attempt SMA in the US are in fact vulnerable to legal threats, arrest, and prosecution [29]. Six states have explicit laws banning SMA (Idaho, Nevada, Arizona, Oklahoma, South Carolina, and Delaware) and there are roughly 40 laws that could be used to prosecute someone who attempts SMA. As of 2019, there have been at least 21 arrests for SMA and criminal investigations in 20 states for alleged SMA [29]. At the time this study was conducted, the online telemedicine organization Women on Web, which provides medication abortion by mail to people in countries where safe abortion is restricted or unavailable, had not yet begun shipping medications to the US through its separate organization Aid Access (Aid Access was launched in 2018). Likely due to the increasing number of state-level abortion restrictions proposed and enacted since 2017, the availability of abortion medications through Aid Access, and the global COVID-19 pandemic, which forced people to minimize in-person clinic visits and led to the temporary elimination of clinic-based abortion services in some states, the number of people seeking and obtaining medication abortion pills online, as well as other forms of SMA, has increased since the fielding of this study. Aid Access reportedly received 21,000 requests for medication within its first year providing the service to people in the US (from March 2018 to March 2019) [16, 30] and nearly 50,000 requests in the period from January 2019 to April 2020 [18].

This study has several limitations. Our findings do not include individuals who attempted SMA with mifepristone or misoprostol, despite previous evidence to suggest that people are self-sourcing these medications for SMA outside of the formal health system [31]. We expect that individuals who use misoprostol on their own to induce an abortion may have different experiences and clinical outcomes compared to those who use other methods for SMA, such as herbs or other over-the-counter medications not specifically indicated for termination of pregnancy. The lack of clinical follow-up of participants in our study prevents the ability to determine whether or not SMA methods used were effective in ending a pregnancy. Collecting data on such a stigmatized, and sometimes illegal, behavior is challenging due to underreporting and fear of disclosure. Those who may have obtained mifepristone or misoprostol outside of the formal healthcare system may have chosen not to participate in this study due to fear of facing legal risks. Our sample includes only adult participants who were comfortable discussing their experiences with SMA and who speak English or Spanish. Three participants reported never having attempted SMA on the telephone despite having reported it previously in the anonymous survey that preceded the interviews. These discrepancies may have been due to errors completing the self-administered survey; confusion between medication abortion, EC, and SMA with misoprostol; or simply a reluctance to talk about personal SMA experiences over the phone.

## Conclusions

We found that people chose SMA over facility-based abortion care because it was convenient, accessible, and private, and that people sought SMA methods that were safe even though they were not always effective. Results from this study provide insight into motivations for seeking SMA as well as the decision-making processes regarding how and when to attempt SMA. Our findings highlight potential differences between SMA attempts that occur before versus after the confirmation of pregnancy via urine or blood test. Those who find themselves in a grey area between pregnancy suspicion and pregnancy confirmation may be more comfortable attempting something on their own without medical assistance. For many interested in SMA as an alternative to facility-based abortion, the window of time during which they believe SMA is worth trying is relatively short and occurs earlier on in pregnancy. People interested in SMA would likely benefit from a medication abortion product that was available over the counter, online, and or in the form of a missed-period pill. Our findings point to the need to expand access to abortion through alternative models of provision so that people can obtain abortions that are safe, effective, private, and accessible.

## Data Availability

The datasets generated and/or analyzed during the current study are not publicly available due to potential confidentiality concerns. Additional information can be made available from the corresponding author on reasonable request.

## Abbreviations

EC: Emergency contraception
SMA: Self-managed abortion
US: United States
